# Mendelian randomization reveals no causal relationship between COVID‐19 susceptibility, hospitalization, or severity and epilepsy

**DOI:** 10.1002/epi4.12818

**Published:** 2023-08-26

**Authors:** Zihua He, Yinghong Li, Shengyi Liu, Jinmei Li

**Affiliations:** ^1^ Department of Neurology, West China Hospital Sichuan University Chengdu China; ^2^ The Department of Neurology Institute of Traditional Chinese Medicine of Sichuan Academy of Chinese Medicine Sciences (Sichuan Second Hospital of T.C.M) Chengdu China

**Keywords:** causal association, COVID‐19, epilepsy, Mendelian randomization

## Abstract

**Objective:**

Observational studies have shown an association between COVID‐19 and epilepsy. However, causality remains unproven. This study aimed to investigate the causative effect of genetically predicted COVID‐19 phenotypes on epilepsy risk using a two‐sample Mendelian randomization (MR) analysis.

**Methods:**

We retrieved summary‐level datasets for three COVID‐19 phenotypes (COVID‐19 susceptibility, COVID‐19 hospitalization, and COVID‐19 severity) and epilepsy from the genome‐wide association studies conducted by the COVID‐19 Host Genetics Initiative (COVID‐19 HGI) and International League Against Epilepsy (ILAE) consortium, respectively. To analyze the final results, nine MR analytic methods were utilized. The inverse‐variance weighted (IVW) method was chosen as the primary approach for data analysis to evaluate the potential causal effect. Other MR analytic methods (MR‐Egger regression, weighted median estimator, mode based‐estimator, and MR‐PRESSO) were used as a supplement to IVW to ensure the robustness of the results.

**Results:**

The IVW approach demonstrated no causal association between any genetically predicted COVID‐19 phenotype and the risk of epilepsy [COVID‐19 susceptibility: odds ratio (OR) = 0.99, 95% confidence interval (CI) = 0.86–1.14, *p* = 0.92; COVID‐19 hospitalization: OR = 1.00, 95% CI = 0.96–1.04, *p* = 0.95; COVID‐19 severity: OR = 0.99, 95% CI = 0.96–1.01, *p* = 0.25]. Other MR complementary methods revealed consistent results. Additionally, no evidence for heterogeneity and horizontal pleiotropy was found.

**Significance:**

This MR study revealed no genetically predicted causal relationship between COVID‐19 phenotypes and epilepsy.

**Key points**

- Mendelian randomization (MR) was used to investigate the causal relationship between genetically predicted COVID‐19 phenotypes and epilepsy.
- This two‐sample MR study did not show a causal relationship between COVID‐19 and epilepsy.
- COVID‐19 may not be a risk factor for epilepsy.

## 1 INTRODUCTION

Since the start of the pandemic, severe acute respiratory syndrome coronavirus 2 (SARS‐CoV‐2), the causative agent of coronavirus disease 2019 (COVID‐19), has been associated with a large number of infections and deaths, representing a major threat to human health and public safety.^1^ As of early July 2023, ~767 million, and more than 6.95 million global cases and deaths of COVID‐19 were estimated, respectively.^2^ Neuropsychiatric manifestations are prevalent among COVID‐19 patients and others who have recovered from the infection, although the virus has been primarily linked to the respiratory system.^3^, ^4^, ^5^ Cognitive impairment and fatigue are common symptoms of post‐COVID‐19 syndrome.^6^ Thus, COVID‐19 severely impacts the brain health of infected individuals, and identifying potential consequences of COVID‐19 may enhance our understanding of its pathogenesis.

About 65–70 million people throughout the world suffer from epilepsy, a neurological illness marked by recurring seizures.^7^, ^8^ Viral infections of the brain are a common cause of epilepsy.^9^ However, it is controversial whether SARS‐CoV‐2 infection is a risk factor for epilepsy. A large retrospective cohort study demonstrated that when matched to comparable influenza patients, COVID‐19 patients had significantly higher rates of both new onset seizures and epilepsy within 6 months following the infection.^10^ Another retrospective cohort study showed that the increased risk of seizures or epilepsy persisted for 2 years after COVID‐19, compared to other respiratory infections.^11^ Similarly, Xu et al. found an increased risk of different neurological conditions, including epilepsy and seizures, in the 12 months following the acute infection phase of COVID‐19.^12^ However, according to a population‐wide study conducted in Sweden, COVID‐19 is not correlated with an increased incidence of epilepsy.^13^ Since the existing data on SARS‐CoV‐2 infection and the risk of epilepsy are derived from observational studies that may be biased by unavoidable confounding factors, it is unknown whether COVID‐19 is causally associated with a higher risk of epilepsy.

Mendelian randomization (MR) is an epidemiological method which utilizes genetic variants as instrumental variables (IVs) to examine the causative association between exposures and outcomes.^14^ The method eliminates confounding bias and is comparable to randomized controlled trials.^14^ In the present study, a two‐sample MR analysis was conducted to evaluate the casual effect of the genetically predicted COVID‐19 phenotypes (COVID‐19 susceptibility, COVID‐19 hospitalization, and COVID‐19 severity) on the risk of epilepsy.

## 2 MATERIALS AND METHODS

### 2.1 Study design

**FIGURE 1:**
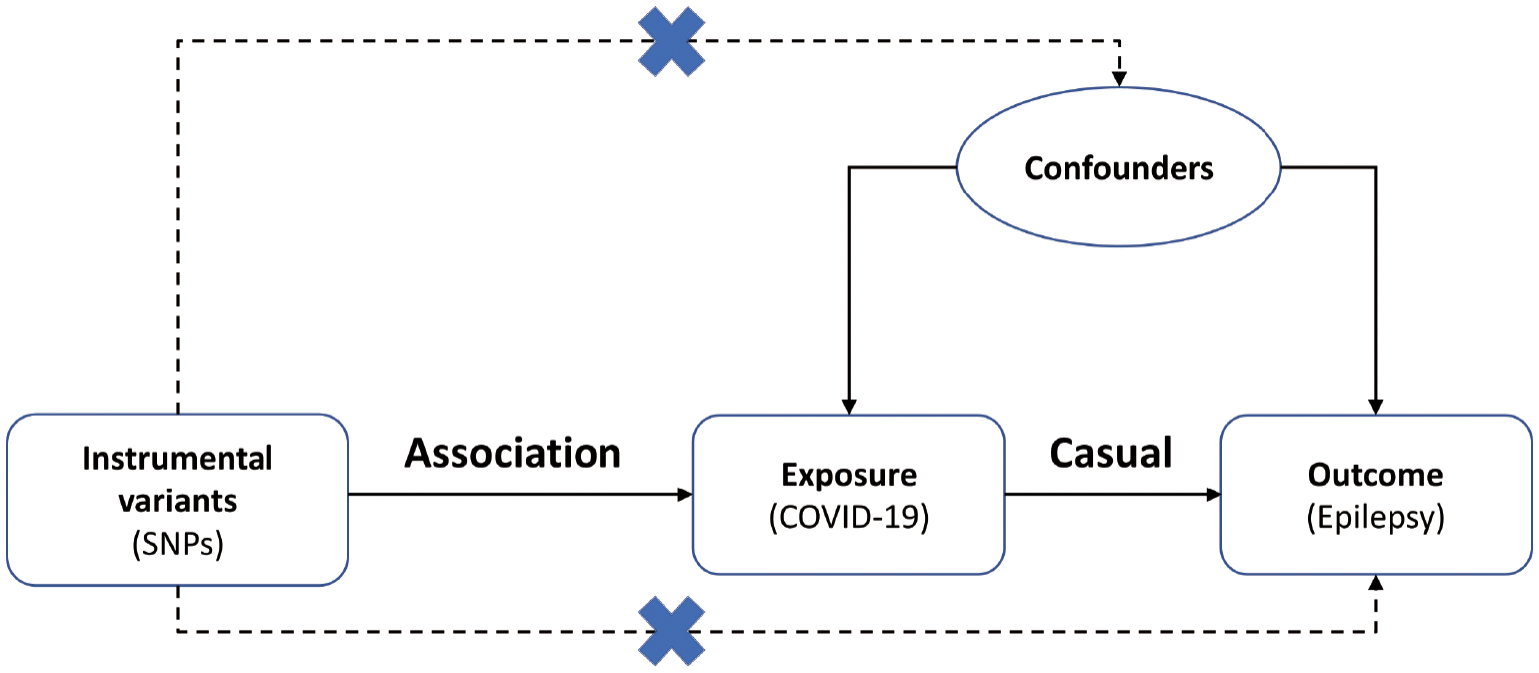
Schematic diagram of the Mendelian randomization (MR) research. SNPs, single nucleotide polymorphisms.

Single nucleotide polymorphisms (SNPs) were chosen from the Genome‐Wide Association Study (GWAS) datasets as the IVs for COVID‐19.^15^ Another GWAS meta‐analysis provides their connection with epilepsy.^16^ A valid genetic instrument is needed to fulfill three key assumptions^17^: it is associated with COVID‐19; it is unrelated to any potential confounders; and it has no effect on epilepsy via factors other than COVID‐19 (i.e., no horizontal pleiotropy; Figure 1). The STROBE‐MR guideline was applied when conducting this investigation.^18^ It should also be noted that all datasets utilized in this work are publicly available, and their original studies already obtained patients' permission and ethical approval. Therefore, no further informed consent or ethical permit was required.

### 2.2 Datasets and IVs selection

Exposure data were obtained from the latest GWAS summary statistics of the COVID‐19 Host Genetics Initiative (COVID‐19 HGI), which includes the susceptibility, hospitalization, and severity of COVID‐19^15^ (www.covid19hg.org, Release 7). RNA RT‐PCR or serologic testing was used as the diagnostic approach for COVID‐19, while patient self‐reported infections and electronic health records were also considered. The susceptibility phenotype was compared between COVID‐19 cases and controls without history of SARS‐CoV‐2 infection. The hospitalization phenotype compared COVID‐19‐hospitalized patients to controls who were not admitted for COVID‐19 or to patients without COVID‐19. The severity phenotype was determined by comparing dead or respiratory‐assisted hospitalized patients with COVID‐19 to individuals that did not have COVID‐19 and others who had the disease but did not have a severe status. Table 1 details characteristics for all GWAS datasets. Additional information about these GWAS is provided in the Appendix S1.

**TABLE 1:** Detailed information of studies and datasets used for analyses.

Data source	Population	Phenotype	Sample size	Cases	h^2^	SE (h^2^)
COVID‐19 HGI	Europeans	COVID‐19 Susceptibility	2 597 856	122 616	0.0020	0.0003
		COVID‐19 Hospitalization	2 095 324	32 519	0.0035	0.0005
		COVID‐19 Severity	1 086 211	13 769	0.0062	0.0012
ILAE	~86% Europeans	Epilepsy	44 889	15 212	0.1067	0.0166

Abbreviations: COVID‐19 HGI, COVID‐19 Host Genetics Initiative; ILAE, International League Against Epilepsy consortium; h^2^, Observed SNP‐heritability calculated using LDSC (see methods); SE (h^2^), Standard errors for h^2^.

GWAS summary statistics of the outcome for all epilepsy were obtained from the International League Against Epilepsy (ILAE) consortium (Table 1).^16^ More details of recruited cohorts, study design, and quality control procedures were described in the original publication.

To select valid IVs, we first identified SNPs which were genome‐wide significant (*p* < 5E‐08) and had a minor allele frequency (MAF) > 0.01. Then, we performed a clumping process (*r* ^2^ < 0.05; region size, 10 Mb) based on the European 1000 Genomes dataset.^19^ For SNPs that were not available in the outcome GWAS, proxy SNPs (*r* ^2^ ≥ 0.8) were obtained from an online website (snipa.helmholtz‐muenchen.de/snipa3/). Moreover, the MR Steiger filtering test was used to identify and remove SNPs that imply reverse directional causality.^20^ Furthermore, to avoid significant correlation of these IVs with potential confounders (*p* < 5E‐08), we searched for the phenotypes associated with variants in Phenoscanner V2.^21^

### 2.3 Statistical analysis

To assess the causal effect of the genetically predicted COVID‐19 phenotypes on epilepsy, we conducted nine MR analytic methods, including inverse‐variance weighted (IVW),^22^ MR‐Egger regression (Bootstrap and general models),^17^ weighted median estimator (Penalized weighted median and weighted median),^23^ mode based‐estimator (simple and weighted modes),^24^ and MR pleiotropy residual sum and outlier (MR‐PRESSO) analytic methods.^25^ As the primary method, we used the IVW fixed‐effect model (IVW‐fe), a conventional MR analysis that results in convincing causal estimates in the absence of pleiotropic effects. We further undertook a series of complementary analytic approaches to evaluate horizontal pleiotropy risk. First, we used the IVW random‐effect model (IVW‐mre), which provides more accurate estimates in the presence of high heterogeneity of SNPs. The MR‐Egger regression was additionally used for detecting the horizontal pleiotropy of IVs and generating the causal estimates.^26^ We further validated the findings through the weighted median estimator, which provides a steady causal assessment when >50% of IVs are valid. Bias can be reduced by the mode based‐estimator, which additionally leads to lower type I error rates, but with a lower accuracy. In addition, we performed the leave‐one‐out, and MR‐PRESSO analyses to identify outlier IVs. Furthermore, heterogeneity evaluation was performed by the Cochran's Q test. In addition to the MR‐Egger regression analyses, the MR‐PRESSO global test was also used to investigate the horizontal pleiotropy. The F‐statistics were calculated to evaluate the strength of each IV.^27^ Finally, we calculated the genetic correlation between COVID‐19 phenotypes and epilepsy using linkage disequilibrium score regression (LDSC). LDSC was also utilized to estimate the SNP heritability for each phenotype based on precomputed LD scores from the 1000 Genomes European reference panel. A Bonferroni‐corrected *p*‐value <0.0167 (0.05/3 exposures) was considered significant. MR analyses were performed with the TwoSampleMR and MR‐PRESSO packages in R software (version 4.1.3), while genetic correlation was calculated with LDSC V1.0.1 (See Appendices S2 and S3).^25^, ^28^, ^29^, ^30^

## 3 RESULTS

The results of MR Steiger filtering tests are presented in Table S1. A total of seven SNPs for COVID‐19 susceptibility, 19 SNPs for COVID‐19 hospitalization, 19 SNPs for COVID‐19 severity were finally included. These SNPs explained more variance in COVID‐19 phenotypes than epilepsy, suggesting causality in the expected direction. Using the PhenoScannerV2 tool, we found that two SNPs were significantly associated with hypertension and alcohol consumption, respectively. Therefore, these were excluded from further analyses. All of the remaining SNPs that were selected as IVs had F‐statistics >10 (18.77–209.67), confirming the robustness of the selected instruments (Table S2). No sample overlap between the COVID‐19 and epilepsy datasets was noticed (Table 1).

**FIGURE 2:**
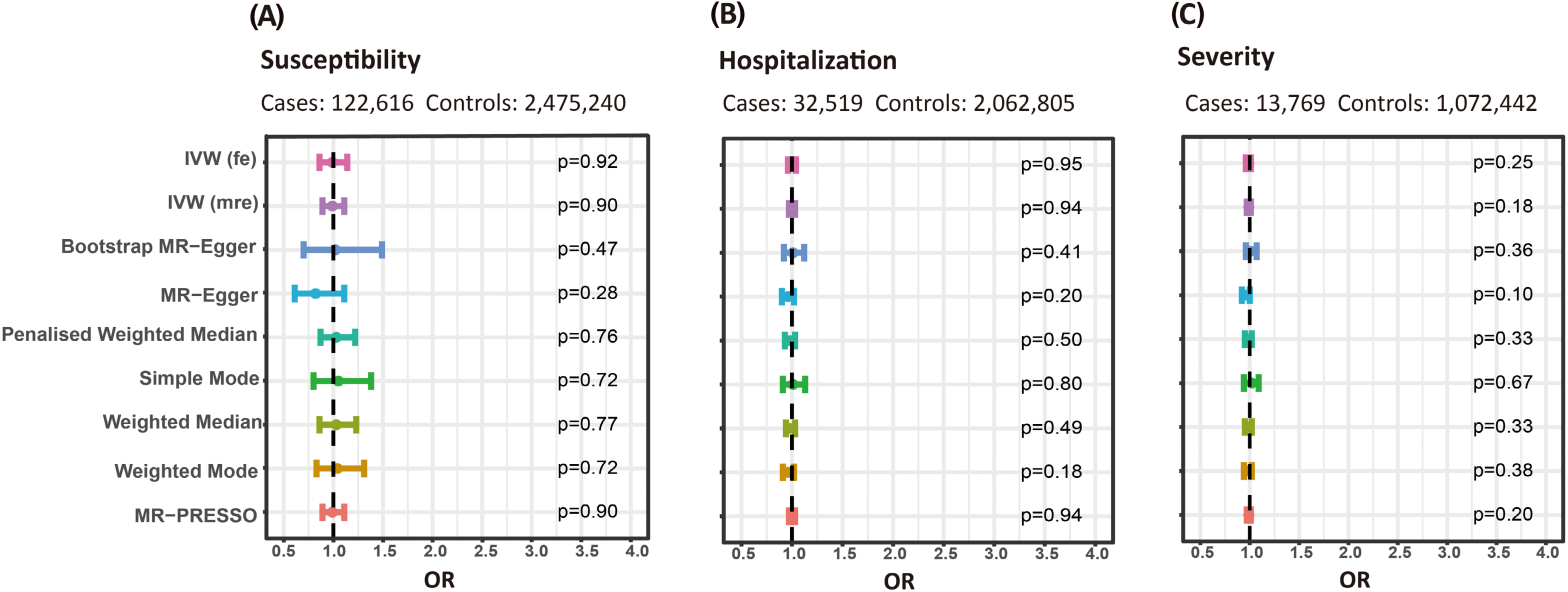
The results of the MR analysis to assess the causative effect of (A) susceptibility, (B) hospitalization, and (C) severity of COVID‐19 on epilepsy. IVW (fe), Inverse variance weighted (fixed‐effect); IVW (mre), Inverse variance weighted (multiplicative random‐effect); MR‐PRESSO, MR Pleiotropy Residual Sum and Outlier; OR, odds ratio.

In the IVW‐fe model, we observed no causal correlation between genetically predicted COVID‐19 phenotypes and epilepsy risk (COVID‐19 susceptibility: odds ratio (OR) = 0.99, 95% confidence interval (CI) = 0.86–1.14, *p* = 0.92; COVID‐19 hospitalization: OR = 1.00, 95%CI = 0.96–1.04, *p* = 0.95; COVID‐19 severity: OR = 0.99, 95%CI = 0.96–1.01, *p* = 0.25; Figure 2). A series of complementary analyses based on different hypotheses confirmed the robustness of the IVW analysis results (Table S3; Figure 2).

**FIGURE 3:**
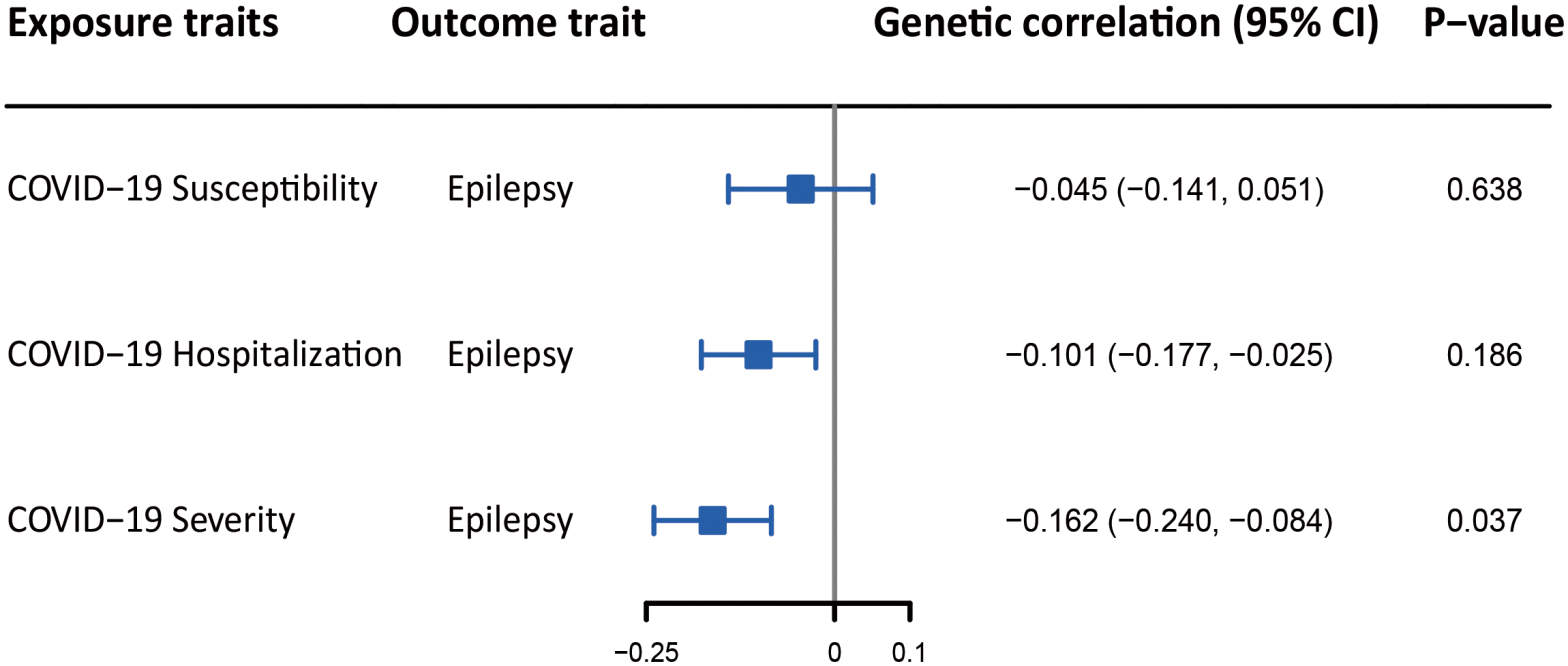
Forest plot showing the genetic correlation between COVID‐19 phenotypes and epilepsy. A Bonferroni‐corrected *p*‐value <0.0167 (0.05/3 exposures) was considered significant.

The Cochran's Q test demonstrated a low degree of heterogeneity among the IVs (Table 2). Using the MR‐PRESSO global test and MR‐Egger intercept terms, no remarkable horizontal pleiotropy was observed (Table 2). Meanwhile, no outliers were detected by the MR‐PRESSO test. Furthermore, the association between genetically predicted COVID‐19 phenotypes and the epilepsy risk was not significantly driven by a single SNP based on the findings of the leave‐one‐out analysis (Figure S1). Genetic correlation analyses revealed a negative association between epilepsy and the three COVID‐19 phenotypes, none of which were significant after correcting for multiple testing (Figure 3).

**TABLE 2:** Heterogeneity and horizontal pleiotropy analyses between COVID‐19 and epilepsy.

Exposure trait	Heterogeneity			Pleiotropy		
	IVW Q	IVW Q df	Cochran's Q *p*	MR‐Egger intercept	MR‐Egger intercept *p*	MR‐PRESSO global test *p*
COVID‐19 Susceptibility	3.27	5	0.66	1.11E‐02	0.25	0.57
COVID‐19 Hospitalization	9.81	17	0.91	5.60E‐03	0.13	0.80
COVID‐19 Severity	13.18	18	0.78	4.89E‐03	0.21	0.80

Abbreviations: df, Cochran's Q test degrees of freedom; IVW, Inverse variance weighted; MR‐Egger, Mendelian randomization‐Egger; MR‐PRESSO, MR‐pleiotropy residual sum and outlier; Q, Cochran's Q test estimate.

## 4 DISCUSSION

The causal effect of the genetically predicted COVID‐19 phenotypes on epilepsy risk was estimated in this study using up to nine MR analytic approaches. The results demonstrated no causative link between genetically predicted COVID‐19 phenotypes and the risk of epilepsy, which was also confirmed by multiple sensitivity analyses. Since SARS‐CoV‐2 infection is associated with a range of neurological sequelae, understanding the casual relationship between COVID‐19 and epilepsy is of great importance for managing this event.

Although some investigations showed a higher risk of epilepsy and seizures post‐COVID‐19, there are multiple probable explanations for this inconsistency. First, certain studies were conducted with both seizures and epilepsy as the outcome.^11^, ^12^ However, early seizures (also known as provoked, insult‐related, or acute symptomatic seizures) are merely symptoms of underlying brain injury and cannot be termed spontaneous seizures.^31^ In contrast, epilepsy is characterized by a persistent propensity to experience recurring unprovoked seizures, and the two are mechanistically distinct.^32^, ^33^ Although early seizures occur in 30% of central nervous system infections, not all individuals with early seizures will develop spontaneous seizures (i.e., epilepsy).^34^, ^35^ For instance, seizures can be caused by fever, hypoxia, and metabolic disturbances in patients with severe COVID‐19.^36^ Certain therapeutics used to treat COVID‐19, such as chloroquine and hydroxychloroquine, also can cause tonic clonic seizures.^37^ Furthermore, our results are similar to those of a Swedish study in which SARS‐CoV‐2 infection was not correlated with a population‐wide elevated incidence of epilepsy.^13^ In observational research, the disparity might also be attributed to reverse causality and unmeasured confounding factors.^23^

Mechanistically, seizures and epilepsy may be a direct result of viral invasion of the central nervous system or an indirect result mediated by the host's immune response.^38^ SARS‐CoV‐2 has been reported to be rarely detected in cerebrospinal fluid.^39^ There was also no evidence of viral invasion in the parenchyma of the frontal lobe and the olfactory bulb of COVID‐19 patients who died during the acute phase.^40^ Results from an autopsy study of COVID‐19 patients revealed that limited indications of inflammation or direct viral cytopathology were observable beyond the respiratory tract, although persistent SARS‐CoV‐2 RNA was detected in the brain.^41^ In addition, Kiran et al. performed autopsies on 41 SARS‐CoV‐2 infected patients and found that detectable levels of virus in the brain were extremely low and had no correlation with histopathological changes, suggesting that the neuropathological manifestations observed in most brains are not caused by direct viral invasion of the brain parenchyma, but rather by systemic inflammation.^42^ Similarly, SARS‐CoV‐2 infected hamsters showed no evidence of viral neuroinvasion.^43^, ^44^ Therefore, the ability of SARS‐CoV‐2 to directly infect brain cells might be limited.^45^

On the other hand, the brains of SARS‐CoV‐2 infected hamsters and patients who died from COVID‐19 exhibited increased blood–brain barrier permeability, expression of interleukin (IL)‐1β and IL‐6, microglial activation, and decreased hippocampal neurogenesis.^43^ However, brain alterations in hamsters were transient.^43^ Another study of cross‐sectional and longitudinal cases revealed that, despite the persistence of cerebral hypermetabolism, the extent and severity of cortical hypometabolism decreased over time, alongside improvements in blood saturation, systemic inflammation, and cognitive performance.^46^ These findings suggest that the cortical dysfunction caused by the synergistic effects of virus‐mediated systemic inflammation and transient hypoxia may be temporary and reversible.^46^ Furthermore, hypoperfusion of cortical cerebral blood flow in patients with severe COVID‐19 showed a longitudinal trend of recovery.^47^ Collectively, seizures after SARS‐CoV‐2 infection might be mediated by transient brain damage and inflammation; however, once these factors are eliminated, alterations in brain structure and function might recover and not contribute to epileptogenesis.

The main advantage of this study is that potential biases, like reverse causality and residual confounders, were reduced by MR analysis compared to traditionally designed observational studies. Secondly, using multiple sensitivity analyses, the robustness of our findings was evaluated with no evidence of heterogeneity or horizontal pleiotropy. The findings of MR Steiger test and Phenoscanner V2 also showed no pleiotropic SNPs. Consequently, the risk of pleiotropic effects in this study was limited.

However, several limitations should be considered. First, the population in this study mainly consisted of Europeans. Therefore, the current conclusions should be carefully interpreted, particularly for non‐European populations. Second, the ability to discern weaker causal links is compromised by the relatively small variance explained by the IVs of the exposures. The correlation between COVID‐19 and epilepsy might become significant in future GWAS summary statistics with larger sample sizes. Third, some cases of COVID‐19 were identified through self‐reporting, which may induce measurement bias. Fourth, the risk of epilepsy in relation to the infection with SARS‐CoV‐2 variants and vaccination could not be investigated due to the limitation of COVID‐19 GWAS data. Fifth, COVID‐19 hospitalization and severity phenotypes might be influenced by factors such as medical conditions in each country that could not be taken into account in the MR analysis. In addition, Due to the linear effect assumption in the MR analysis, we were unable to assess nonlinear associations between COVID‐19 exposures and the risk of epilepsy. Finally, even though this study indicates that COVID‐19 exposures are not causally associated with epilepsy, it is important to note that the MR analysis is an unvalidated prediction. Therefore, further investigation and validation is still warranted.

## 5 CONCLUSION

In summary, this two‐sample MR investigation did not show the genetically predicted causal association between COVID‐19 phenotypes and an increased risk of epilepsy in the European population. Future MR studies based on larger sample sizes of GWAS data require additional research on this topic.

## AUTHOR CONTRIBUTIONS

Study conception, design, and draft preparation: Z.H. and Y.L.; Data collection and analyses: Z.H., Y.L., and S.L.; Supervision of the study and revision of the manuscript: J.L.

## CONFLICT OF INTEREST STATEMENT

The authors declare that the research was conducted in the absence of any commercial or financial relationships that could be construed as a potential conflict of interest. We confirm that we have read the Journal's position on issues involved in ethical publication and affirm that this report is consistent with those guidelines.

## ETHICS STATEMENT

All datasets utilized in this work are publicly available, and their original studies already obtained patients' permissions and ethical approval. No further informed consent or ethical permit was required.

## Supporting information


Figure S1.

Table S1.

Table S2.

Table S3.

Appendix S1–S3:

## Data Availability

All data used in this study are publicly available. GWAS summary data for COVID‐19 and epilepsy were obtained from https://www.covid19hg.org/results/r7/ and https://gwas.mrcieu.ac.uk/, respectively.
