# Supplementary material for: Mendelian randomization reveals no causal relationship between COVID‐19 susceptibility, hospitalization, or severity and epilepsy
Source: Epilepsia Open. 2023 Aug 26;8(4):1452–9. doi: 10.1002/epi4.12818 (PMC10690698; doi:10.1002/epi4.12818)
Supplement: Supplementary file 1 — Figure S1. [file EPI4-8-1452-s003.docx]

**Figure S1: Mendelian randomization analysis results for COVID-19 on the risk of epilepsy.**





Column 1: Scatter plot showing the MR estimates for the relationship between COVID-19 and epilepsy risk. Column 2: Funnel plot illustrating the overall heterogeneity of the MR estimates for the effect of COVID-19 on epilepsy. Column 3: Forest plot of leave-one-out sensitivity analysis for COVID-19 on epilepsy risk.
