# Supplementary material for: Mendelian randomization reveals no causal relationship between COVID‐19 susceptibility, hospitalization, or severity and epilepsy
Source: Epilepsia Open. 2023 Aug 26;8(4):1452–9. doi: 10.1002/epi4.12818 (PMC10690698; doi:10.1002/epi4.12818)
Supplement: Supplementary file 2 — Table S1. [file EPI4-8-1452-s005.docx]

| Table S1. Results of the MR Steiger direction test. | | |  |  |  |  |
| --- | --- | --- | --- | --- | --- | --- |
| **Exposure Trait** | **SNP** | **rsq.exposure** | **rsq.outcome** | **steiger_dir^*^** | **steiger_pval** |  |
| COVID-19 Susceptibility | rs1123573 | 1.18E-05 | 1.50E-07 | TRUE | 0.522 |  |
|  | rs17367421 | 1.25E-05 | 7.11E-06 | TRUE | 0.856 |  |
|  | rs2290859 | 4.21E-05 | 9.64E-07 | TRUE | 0.247 |  |
|  | rs2834158 | 2.67E-05 | 2.35E-05 | TRUE | 0.946 |  |
|  | rs35044562 | 8.77E-05 | 3.96E-05 | TRUE | 0.518 |  |
|  | rs4801778 | 2.13E-05 | 1.78E-06 | TRUE | 0.491 |  |
|  | **rs554833** | 1.40E-04 | 3.08E-05 | TRUE | 0.187 |  |
| COVID-19 Hospitalization | rs1123573 | 2.08E-05 | 1.50E-07 | TRUE | 0.382 |  |
|  | rs113098443 | 3.23E-05 | 2.82E-05 | TRUE | 0.939 |  |
|  | rs11579758 (proxy for rs3014983) | 1.60E-05 | 8.26E-06 | TRUE | 0.813 |  |
|  | rs117169628 | 2.62E-05 | 1.90E-06 | TRUE | 0.433 |  |
|  | rs12329760 (proxy for rs915823) | 1.43E-05 | 1.05E-05 | TRUE | 0.910 |  |
|  | rs1392288 | 2.96E-05 | 9.15E-06 | TRUE | 0.612 |  |
|  | rs17412601 | 2.09E-05 | 1.37E-06 | TRUE | 0.476 |  |
|  | rs2075741 | 1.46E-05 | 1.43E-05 | TRUE | 0.993 |  |
|  | rs2102497 | 1.43E-05 | 3.30E-06 | TRUE | 0.680 |  |
|  | rs25884 | 1.56E-05 | 5.73E-06 | TRUE | 0.744 |  |
|  | rs2897075 | 1.68E-05 | 2.06E-06 | TRUE | 0.576 |  |
|  | **rs63750417** | 3.05E-05 | 8.04E-09 | TRUE | 0.255 |  |
|  | rs657152 | 5.01E-05 | 4.62E-05 | TRUE | 0.952 |  |
|  | rs6778422 | 1.83E-05 | 8.89E-11 | TRUE | 0.371 |  |
|  | rs67959919 | 3.76E-04 | 4.72E-05 | TRUE | 0.009 |  |
|  | rs6992869 (proxy for rs2326562) | 1.42E-05 | 5.57E-08 | TRUE | 0.459 |  |
|  | rs78314212 | 2.41E-05 | 1.03E-07 | TRUE | 0.336 |  |
|  | rs9577395 (proxy for rs12585036) | 3.30E-05 | 1.72E-05 | TRUE | 0.738 |  |
|  | rs9636867 | 7.58E-05 | 2.29E-05 | TRUE | 0.411 |  |
| COVID-19 Severity | rs10066378 | 2.90E-05 | 1.82E-05 | TRUE | 0.816 |  |
|  | rs11208559 | 3.30E-05 | 9.67E-06 | TRUE | 0.585 |  |
|  | rs1123573 | 4.49E-05 | 1.50E-07 | TRUE | 0.190 |  |
|  | rs114427537 | 3.46E-05 | 1.65E-05 | TRUE | 0.705 |  |
|  | rs117169628 | 5.66E-05 | 1.90E-06 | TRUE | 0.202 |  |
|  | rs12613936 (proxy for rs12614007) | 2.86E-05 | 1.56E-06 | TRUE | 0.395 |  |
|  | rs17713054 | 7.77E-04 | 3.50E-05 | TRUE | 0.000 |  |
|  | rs2075741 | 4.35E-05 | 1.43E-05 | TRUE | 0.559 |  |
|  | rs2236645 | 4.76E-05 | 1.55E-06 | TRUE | 0.241 |  |
|  | rs2897075 | 3.45E-05 | 2.06E-06 | TRUE | 0.357 |  |
|  | rs343320 | 2.89E-05 | 1.25E-07 | TRUE | 0.297 |  |
|  | rs41264915 | 7.47E-05 | 7.36E-05 | TRUE | 0.990 |  |
|  | rs61882275 | 6.81E-05 | 4.22E-05 | TRUE | 0.716 |  |
|  | rs62244824 | 6.66E-05 | 3.66E-05 | TRUE | 0.661 |  |
|  | rs6778422 | 3.88E-05 | 8.89E-11 | TRUE | 0.197 |  |
|  | rs77534576 | 4.94E-05 | 2.95E-05 | TRUE | 0.739 |  |
|  | rs9305744 | 3.06E-05 | 5.35E-08 | TRUE | 0.271 |  |
|  | rs9577395 (proxy for rs12585036) | 6.20E-05 | 1.72E-05 | TRUE | 0.439 |  |
|  | rs9636867 | 1.37E-04 | 2.29E-05 | TRUE | 0.151 |  |
| ^*^SNPs with “TRUE” MR Steiger results suggest causality in the expected direction (ie, thoses explaining more variance in exposure than in outcome). A bolded SNP means that the SNP is associated with potential confounders (p < 5 × 10-8) will be removed from the study. | | | | | |  |
|  |  |  |  |  |  |  |
|  |  |  |  |  |  |  |
