# Supplementary material for: Mendelian randomization reveals no causal relationship between COVID‐19 susceptibility, hospitalization, or severity and epilepsy
Source: Epilepsia Open. 2023 Aug 26;8(4):1452–9. doi: 10.1002/epi4.12818 (PMC10690698; doi:10.1002/epi4.12818)
Supplement: Supplementary file 3 — Table S2. [file EPI4-8-1452-s004.docx]

| Table S2. SNPs used as genetic instruments in each analysis. | | | | | | | | | | |  |
| --- | --- | --- | --- | --- | --- | --- | --- | --- | --- | --- | --- |
| **Exposure Trait** | **SNP** | **Chr:pos** | **EA** | **OA** | **EAF** | **Beta** | **SE** | **R^2^ (%)** | ***F*** | **P-Value** |  |
| COVID-19 Susceptibility | rs1123573 | 2:60707588 | G | A | 0.3789 | -0.0264 | 0.0048 | 0.00807 | 209.67 | 3.16E-08 |  |
|  | rs17367421 | 1:155287241 | C | G | 0.0505 | -0.0635 | 0.0112 | 0.00345 | 89.57 | 1.27E-08 |  |
|  | rs2290859 | 3:101525625 | T | C | 0.3510 | -0.0508 | 0.0049 | 0.00792 | 205.74 | 1.32E-25 |  |
|  | rs2834158 | 21:34617213 | C | T | 0.6611 | -0.0405 | 0.0049 | 0.00790 | 205.29 | 8.51E-17 |  |
|  | rs35044562 | 3:45909024 | G | A | 0.0776 | 0.1280 | 0.0085 | 0.00454 | 117.98 | 1.64E-51 |  |
|  | rs4801778 | 19:49370609 | T | G | 0.1771 | -0.0448 | 0.0060 | 0.00638 | 165.75 | 1.07E-13 |  |
| COVID-19 Hospitalization | rs1123573 | 2:60707588 | G | A | 0.3773 | -0.0688 | 0.0104 | 0.00457 | 95.86 | 4.13E-11 |  |
|  | rs113098443 | 3:46374798 | C | A | 0.4566 | -0.0784 | 0.0095 | 0.00500 | 104.85 | 2.02E-16 |  |
|  | rs11579758 (proxy for rs3014983) | 1:65445542 | A | G | 0.7360 | -0.0695 | 0.0120 | 0.00398 | 83.31 | 6.99E-09 |  |
|  | rs117169628 | 16:89262657 | A | G | 0.1440 | 0.1011 | 0.0137 | 0.00350 | 73.25 | 1.27E-13 |  |
|  | rs12329760 (proxy for rs915823) | 21:42852497 | T | C | 0.2375 | -0.0685 | 0.0125 | 0.00382 | 79.97 | 4.33E-08 |  |
|  | rs1392288 | 3:46034791 | C | T | 0.7425 | 0.0878 | 0.0111 | 0.00428 | 89.72 | 3.33E-15 |  |
|  | rs17412601 | 3:101499275 | C | T | 0.3513 | -0.0683 | 0.0103 | 0.00463 | 96.93 | 3.69E-11 |  |
|  | rs2075741 | 19:10401098 | C | G | 0.4340 | -0.0523 | 0.0095 | 0.00504 | 105.57 | 3.3E-08 |  |
|  | rs2102497 | 16:54255222 | C | T | 0.7339 | 0.0644 | 0.0117 | 0.00406 | 85.16 | 4.21E-08 |  |
|  | rs25884 | 5:131412238 | G | A | 0.2249 | 0.0669 | 0.0117 | 0.00408 | 85.45 | 1.1E-08 |  |
|  | rs2897075 | 7:99630342 | T | C | 0.3740 | 0.0585 | 0.0099 | 0.00484 | 101.45 | 2.88E-09 |  |
|  | rs657152 | 9:136139265 | A | C | 0.3864 | 0.1001 | 0.0098 | 0.00489 | 102.41 | 1.18E-24 |  |
|  | rs6778422 | 3:46590552 | A | G | 0.1119 | 0.1020 | 0.0165 | 0.00290 | 60.74 | 5.91E-10 |  |
|  | rs67959919 | 3:45871908 | A | G | 0.0774 | 0.4916 | 0.0175 | 0.00273 | 57.10 | 2.36E-173 |  |
|  | rs6992869 (proxy for rs2326562) | 8:61395832 | C | T | 0.3814 | 0.0531 | 0.0097 | 0.00490 | 102.71 | 4.81E-08 |  |
|  | rs78314212 | 21:35312916 | T | C | 0.0849 | 0.1220 | 0.0172 | 0.00278 | 58.22 | 1.22E-12 |  |
|  | rs9577395 (proxy for rs12585036) | 13:113534984 | G | C | 0.2163 | 0.0967 | 0.0116 | 0.00410 | 85.93 | 9.39E-17 |  |
|  | rs9636867 | 21:34609944 | G | A | 0.3357 | 0.1282 | 0.0102 | 0.00469 | 98.27 | 2.05E-36 |  |
| COVID-19 Severity | rs10066378 | 5:131776967 | C | T | 0.1159 | 0.1180 | 0.0210 | 0.00438 | 47.61 | 1.96E-08 |  |
|  | rs11208559 | 1:65435283 | G | C | 0.2912 | 0.1025 | 0.0171 | 0.00537 | 58.37 | 2.17E-09 |  |
|  | rs1123573 | 2:60707588 | G | A | 0.3702 | -0.1062 | 0.0152 | 0.00606 | 65.79 | 2.8E-12 |  |
|  | rs114427537 | 1:156081542 | T | C | 0.0196 | 0.3266 | 0.0533 | 0.00173 | 18.77 | 8.8E-10 |  |
|  | rs117169628 | 16:89262657 | A | G | 0.1370 | 0.1575 | 0.0201 | 0.00459 | 49.81 | 4.36E-15 |  |
|  | rs12613936 (proxy for rs12614007) | 2:57316415 | T | C | 0.7494 | 0.0941 | 0.0169 | 0.00545 | 59.24 | 2.46E-08 |  |
|  | rs17713054 | 3:45859651 | A | G | 0.0746 | 0.7558 | 0.0260 | 0.00354 | 38.45 | 1.09E-185 |  |
|  | rs2075741 | 19:10401098 | C | G | 0.4280 | -0.0955 | 0.0139 | 0.00662 | 71.96 | 6.37E-12 |  |
|  | rs2236645 | 21:35342238 | T | C | 0.0848 | 0.1794 | 0.0250 | 0.00369 | 40.08 | 6.46E-13 |  |
|  | rs2897075 | 7:99630342 | T | C | 0.3757 | 0.0880 | 0.0144 | 0.00640 | 69.57 | 9.32E-10 |  |
|  | rs343320 | 3:146234909 | A | G | 0.0700 | 0.1542 | 0.0275 | 0.00335 | 36.35 | 2.06E-08 |  |
|  | rs41264915 | 1:155167786 | G | A | 0.0944 | -0.2063 | 0.0229 | 0.00402 | 43.68 | 2.09E-19 |  |
|  | rs61882275 | 11:34504292 | A | G | 0.3432 | -0.1261 | 0.0147 | 0.00628 | 68.22 | 8.08E-18 |  |
|  | rs62244824 | 3:46361367 | G | T | 0.4466 | -0.1196 | 0.0141 | 0.00655 | 71.10 | 1.8E-17 |  |
|  | rs6778422 | 3:46590552 | A | G | 0.1174 | 0.1580 | 0.0243 | 0.00378 | 41.10 | 8.4E-11 |  |
|  | rs77534576 | 17:47940666 | T | C | 0.0339 | 0.3109 | 0.0424 | 0.00217 | 23.57 | 2.33E-13 |  |
|  | rs9305744 | 21:42842988 | A | G | 0.2679 | -0.0993 | 0.0172 | 0.00534 | 58.03 | 8.27E-09 |  |
|  | rs9577395 (proxy for rs12585036) | 13:113534984 | G | C | 0.2133 | 0.1414 | 0.0172 | 0.00534 | 58.01 | 2.32E-16 |  |
|  | rs9636867 | 21:34609944 | G | A | 0.3312 | 0.1839 | 0.0151 | 0.00610 | 66.28 | 3.47E-34 |  |
| SNP, single-nucleotide polymorphism; Chr, chromosome; pos, position; EA, effect allele; OA, other allele; R^2^, percentage of the variation of exposure trait explained by the SNP; *F*, *F* statistic; Beta, estimate of the effect of the association; SE, standard error. | | | | | | | | | | |  |
|  |  |  |  |  |  |  |  |  |  |  |  |
|  |  |  |  |  |  |  |  |  |  |  |  |
