# Supplementary material for: Mendelian randomization reveals no causal relationship between COVID‐19 susceptibility, hospitalization, or severity and epilepsy
Source: Epilepsia Open. 2023 Aug 26;8(4):1452–9. doi: 10.1002/epi4.12818 (PMC10690698; doi:10.1002/epi4.12818)
Supplement: Supplementary file 4 — Table S3. [file EPI4-8-1452-s001.docx]

| Table S3. Mendelian randomization results. | |  |  |  |  |  |  |  |  |  |  |  |  |  |  |  |  |  |
| --- | --- | --- | --- | --- | --- | --- | --- | --- | --- | --- | --- | --- | --- | --- | --- | --- | --- | --- |
| **Exposure Trait** | **IVW (fe)** |  | **IVW (mre)** |  | **Bootstrap MR-Egger** | | **MR-Egger** |  | **Penalized Weighted Median** | | **Simple Mode** |  | **Weighted Median** | | **Weighted Mode** |  | **MR-PRESSO** |  |
|  | **OR (95% CI)** | **P-Value** | **OR (95% CI)** | **P-Value** | **OR (95% CI)** | **P-Value** | **OR (95% CI)** | **P-Value** | **OR (95% CI)** | **P-Value** | **OR (95% CI)** | **P-Value** | **OR (95% CI)** | **P-Value** | **OR (95% CI)** | **P-Value** | **OR (95% CI)** | **P-Value** |
| COVID-19 Susceptibility | 0.99 (0.86, 1.14) | 0.92 | 0.99 (0.89, 1.11) | 0.90 | 1.02 (0.7, 1.49) | 0.47 | 0.82 (0.61, 1.11) | 0.28 | 1.03 (0.87, 1.22) | 0.76 | 1.05 (0.8, 1.38) | 0.72 | 1.03 (0.86, 1.23) | 0.77 | 1.04 (0.83, 1.31) | 0.72 | 0.99 (0.89, 1.11) | 0.90 |
| COVID-19 Hospitalization | 1.00 (0.96, 1.04) | 0.95 | 1.00 (0.97, 1.03) | 0.94 | 1.01 (0.92, 1.12) | 0.41 | 0.96 (0.9, 1.02) | 0.20 | 0.98 (0.93, 1.03) | 0.50 | 1.01 (0.91, 1.13) | 0.80 | 0.98 (0.94, 1.03) | 0.49 | 0.96 (0.91, 1.02) | 0.18 | 1.00 (0.97, 1.03) | 0.94 |
| COVID-19 Severity | 0.99 (0.96, 1.01) | 0.25 | 0.99 (0.97, 1.01) | 0.18 | 1.01 (0.96, 1.07) | 0.36 | 0.96 (0.92, 1.00) | 0.10 | 0.98 (0.95, 1.02) | 0.33 | 1.02 (0.94, 1.09) | 0.67 | 0.98 (0.95, 1.02) | 0.33 | 0.98 (0.94, 1.02) | 0.38 | 0.99 (0.97, 1.01) | 0.20 |
| CI, confidence interval; OR, odds ratio; IVW (fe), Inverse-variance weighted (fixed-effect); IVW (mre), Inverse variance weighted (multiplicative random-effect); MR-PRESSO, MR Pleiotropy Residual Sum and Outlier. | | | | | | | | | | | | |  |  |  |  |  |  |
