# Supplementary material for: Mendelian randomization reveals no causal relationship between COVID‐19 susceptibility, hospitalization, or severity and epilepsy
Source: Epilepsia Open. 2023 Aug 26;8(4):1452–9. doi: 10.1002/epi4.12818 (PMC10690698; doi:10.1002/epi4.12818)
Supplement: Supplementary file 5 — Appendix S1–S3: [file EPI4-8-1452-s002.docx]

**Supplementary Information 1. Description of the GWAS summary data.**

1. **COVID-19**

The COVID-19 GWAS (Genome-wide Association Study) used in this study was performed by the COVID-19 Host Genetics Initiative (COVID-19 HGI), which contains three phenotypes: susceptibility, hospitalization, and severity of COVID-19 ^1^. Each contributing study independently genotyped the samples and performed quality controls, data imputation, and analysis, while adhering to COVID-19 HGI guidelines ^2^. For single-variant and gene-based association analyses, the SAIGE R software is recommended ^3^. Meta-analysis of the summary statistics for specific studies was then conducted. GWAS summary statistics from European populations were utilized in our study (COVID-19 susceptibility: 122,616 cases and 2,475,240 controls; COVID-19 hospitalization: 32,519 cases and 2,062,805 controls; COVID-19 severity: 13,769 cases and 1,072,442 controls), which are available at covid19hg.org/results/r7/.

**2. Epilepsy**

The epilepsy GWAS utilized in this investigation was performed by the International League Against Epilepsy (ILAE) Consortium and included 15,212 epilepsy cases and 29,677 controls (~86% Europeans) ^4^. Epilepsy was diagnosed by epileptologists according to magnetic resonance imaging, electroencephalography and clinical history. Epilepsy cases were categorized into three major subtypes: focal epilepsy (9,671 cases), genetic generalized epilepsy (3,769 cases), and unclassified epilepsy (1,772 cases). All subjects were assigned to three main ancestry groups (European, Asian and African-American) based on the results of genotype-based principal component analysis. Association analyses for each SNP were conducted using a linear mixed model in BOLT-LMM and combined with the trans-ethnic meta-analysis. The GWAS summary statistics for epilepsy used in this investigation are available at gwas.mrcieu.ac.uk/datasets/ieu-b-8/ and epigad.org/gwas_ilae2018_16loci.html.

**Supplementary Information 2. Estimation of the causal relationship between COVID-19 phenotypes and epilepsy.**

1. **Instrumental variables selection**

We estimated the causal relationship between COVID-19 phenotypes and epilepsy using the "TwoSampleMR" package (github.com/MRCIEU/TwoSampleMR) ^5^, based on the GWAS summary statistics. Using the "read_exposure_data" function, the GWAS summary statistics for COVID-19 phenotypes were read. We chose the SNPs with p-values < 5E-08 and minor allele frequencies (MAF) > 0.01 and then used the "clump_data" function (the parameter "clump_r2" was set to 0.05, the parameter "clump_kb" was set to 10000) to perform the clumping process. Then, we extracted the aforementioned SNPs from the epilepsy GWAS summary statistics stored in the IEU database (gwas.mrcieu.ac.uk/) using the "extract_outcome_data" function. A website (snipa.helmholtz-muenchen.de/snipa3/) was used to find substitutes for the SNPs that were not included in the outcome GWAS data but included in the exposure GWAS data. Using the "harmonise_data" function, we harmonized the effects of the SNPs on outcome and exposure relative to the same allele. Moreover, "steiger_filtering" function was used to exclude the SNPs with reverse causality. Using phenoscannerV2 ([www.phenoscanner.medschl.cam.ac.uk](http://www.phenoscanner.medschl.cam.ac.uk)) ^6^, we further screened the SNPs that were retained in the previous step. Only SNPs unrelated to potential confounders were selected as instrumental variables (IVs) for MR analysis.

1. **Sensitivity, heterogeneity, and horizontal pleiotropy tests**

We used the "mr_heterogeneity" function to detect the heterogeneity of the selected IVs. Using the "mr_pleiotropy_test" function, horizontal pleiotropy tests were conducted. The "mr_leaveoneout" function was used to perform the leave one out sensitivity analysis. Each IV was subjected to a separate 2-sample MR using the "mr_singlesnp" function, and funnel and scatter plots were generated using the "mr_funnel_plot" and "mr_forest_plot" functions, respectively. In addition, the Mendelian Randomization Pleiotropy Residual Sum and Outlier (MR-PRESSO) test was conducted utilizing the "mr_presso" function of the "MRPRESSO" R package (github.com/rondolab/MRPRESSO) ^7^.

1. **MR analysis**

Using the "mr" function, IVs were utilized to perform MR tests. The "method_list" parameter was set to "mr_ivw_fe", "mr_ivw_mre", "mr_egger_regression_bootstrap", "mr_egger_regression", "mr_penalised_weighted_median", "mr_simple_mode", "mr_weighted_median", and "mr_weighted_mode", which represented the methods we used in the MR analysis. We utilized the "generate_odds_ratios" function to calculate the odds ratio (OR) and 95% confidence intervals (CI) based on the results of the "mr" and "mr_presso" functions. We then extracted the p-value, OR, and 95% CI from the MR tests and visualized them using the "ggplot2" R package (ggplot2.tidyverse.org). We also plotted the scatter plots of the results of the Inverse variance weighted (fixed effects) method and the weighted median method using the "mr_scatter_plot" function.

**Supplementary Information 3. Calculation of the genetic correlation between COVID-19 phenotypes and epilepsy.**

On the basis of the GWAS summary statistics, we calculated the genetic correlation between COVID-19 phenotypes and epilepsy using linkage disequilibrium score regression (LDSC, github.com/bulik/ldsc) ^8, 9^. The LD (linkage disequilibrium) scores utilized in the present study were pre-computed using the 1000 Genomes European data ^8^. Then, we used the "munge_sumstats.py" script to convert the GWAS summary statistics into "ldsc" format. The "ldsc.py" script was utilized to calculate the genetic correlation between the three exposure data and the outcome data.

**References**

1. The COVID-19 Host Genetics Initiative, a global initiative to elucidate the role of host genetic factors in susceptibility and severity of the SARS-CoV-2 virus pandemic Eur J Hum Genet. 2020 Jun;28:715-718.

2. Mapping the human genetic architecture of COVID-19 Nature. 2021 Dec;600:472-477.

3. Zhou W, Nielsen JB, Fritsche LG, Dey R, Gabrielsen ME, Wolford BN, et al. Efficiently controlling for case-control imbalance and sample relatedness in large-scale genetic association studies Nat Genet. 2018 Sep;50:1335-1341.

4. International League Against Epilepsy Consortium on Complex E. Genome-wide mega-analysis identifies 16 loci and highlights diverse biological mechanisms in the common epilepsies Nat Commun. 2018 Dec 10;9:5269.

5. Hemani G, Zheng J, Elsworth B, Wade KH, Haberland V, Baird D, et al. The MR-Base platform supports systematic causal inference across the human phenome Elife. 2018 May 30;7.

6. Kamat MA, Blackshaw JA, Young R, Surendran P, Burgess S, Danesh J, et al. PhenoScanner V2: an expanded tool for searching human genotype-phenotype associations Bioinformatics. 2019 Nov 1;35:4851-4853.

7. Verbanck M, Chen CY, Neale B, Do R. Detection of widespread horizontal pleiotropy in causal relationships inferred from Mendelian randomization between complex traits and diseases Nat Genet. 2018 May;50:693-698.

8. Bulik-Sullivan BK, Loh PR, Finucane HK, Ripke S, Yang J, Patterson N, et al. LD Score regression distinguishes confounding from polygenicity in genome-wide association studies Nat Genet. 2015 Mar;47:291-295.

9. Bulik-Sullivan B, Finucane HK, Anttila V, Gusev A, Day FR, Loh PR, et al. An atlas of genetic correlations across human diseases and traits Nat Genet. 2015 Nov;47:1236-1241.
